# Supplementary figures and images for: Phylogenetic analysis of higher-level relationships within Hydroidolina (Cnidaria: Hydrozoa) using mitochondrial genome data and insight into their mitochondrial transcription
Source: PeerJ. 2015 Nov 19;3:e1403. doi: 10.7717/peerj.1403 (PMC4655093; doi:10.7717/peerj.1403)

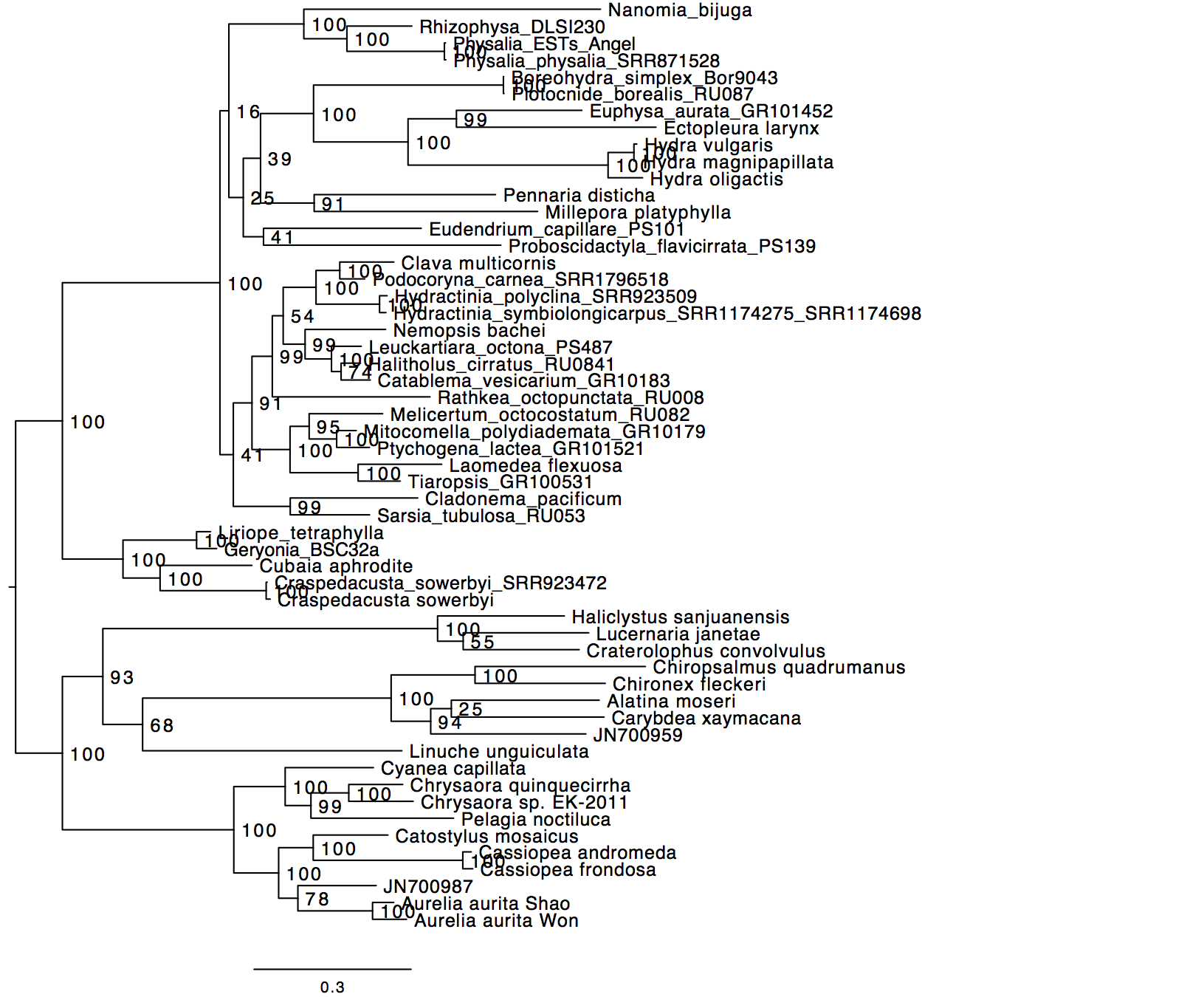

Supplement: Figure S1 — Support values correspond to bootstrap values. [file peerj-03-1403-s001.jpg]

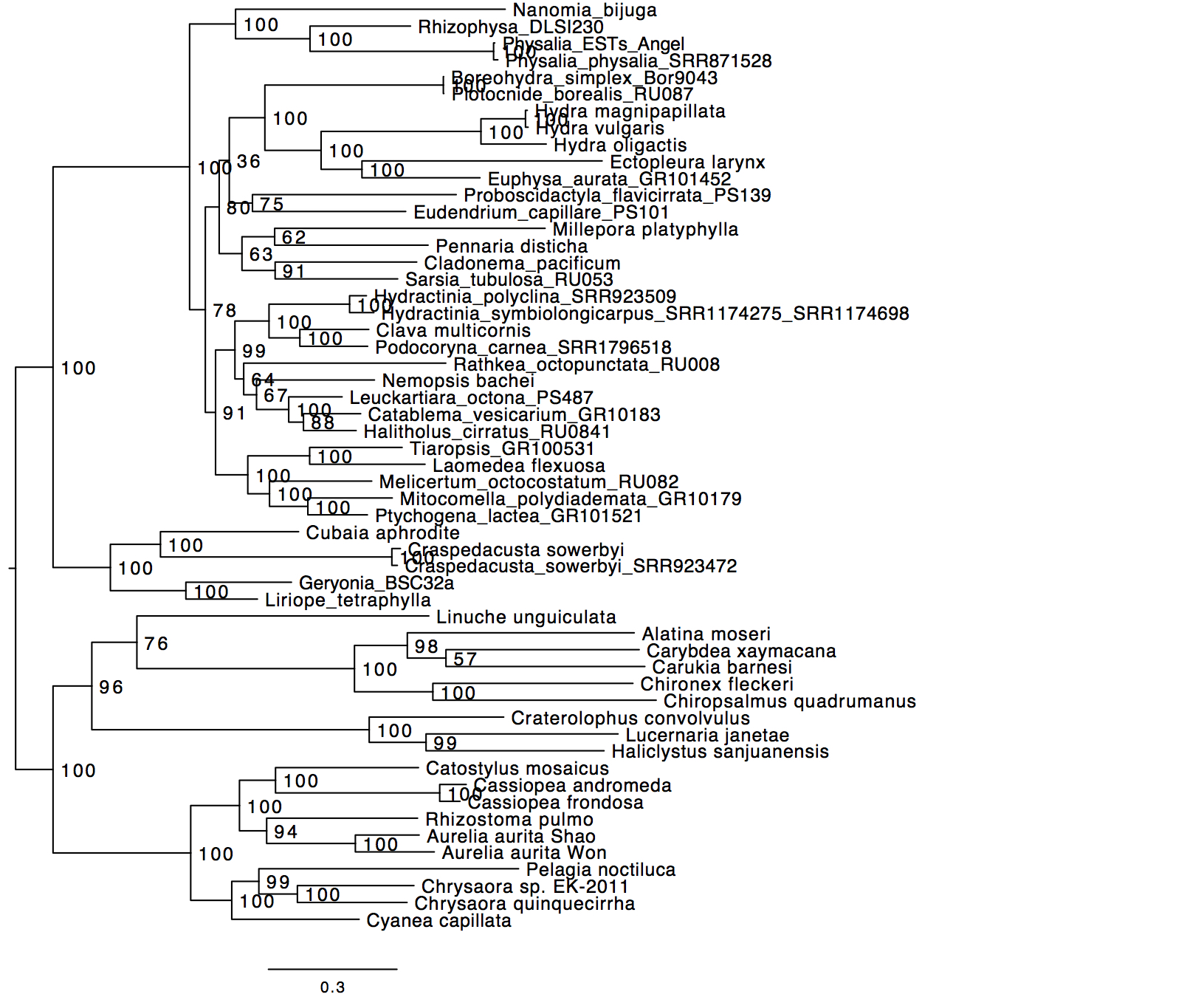

Supplement: Figure S2 — Support values correspond to bootstrap values. [file peerj-03-1403-s002.jpg]

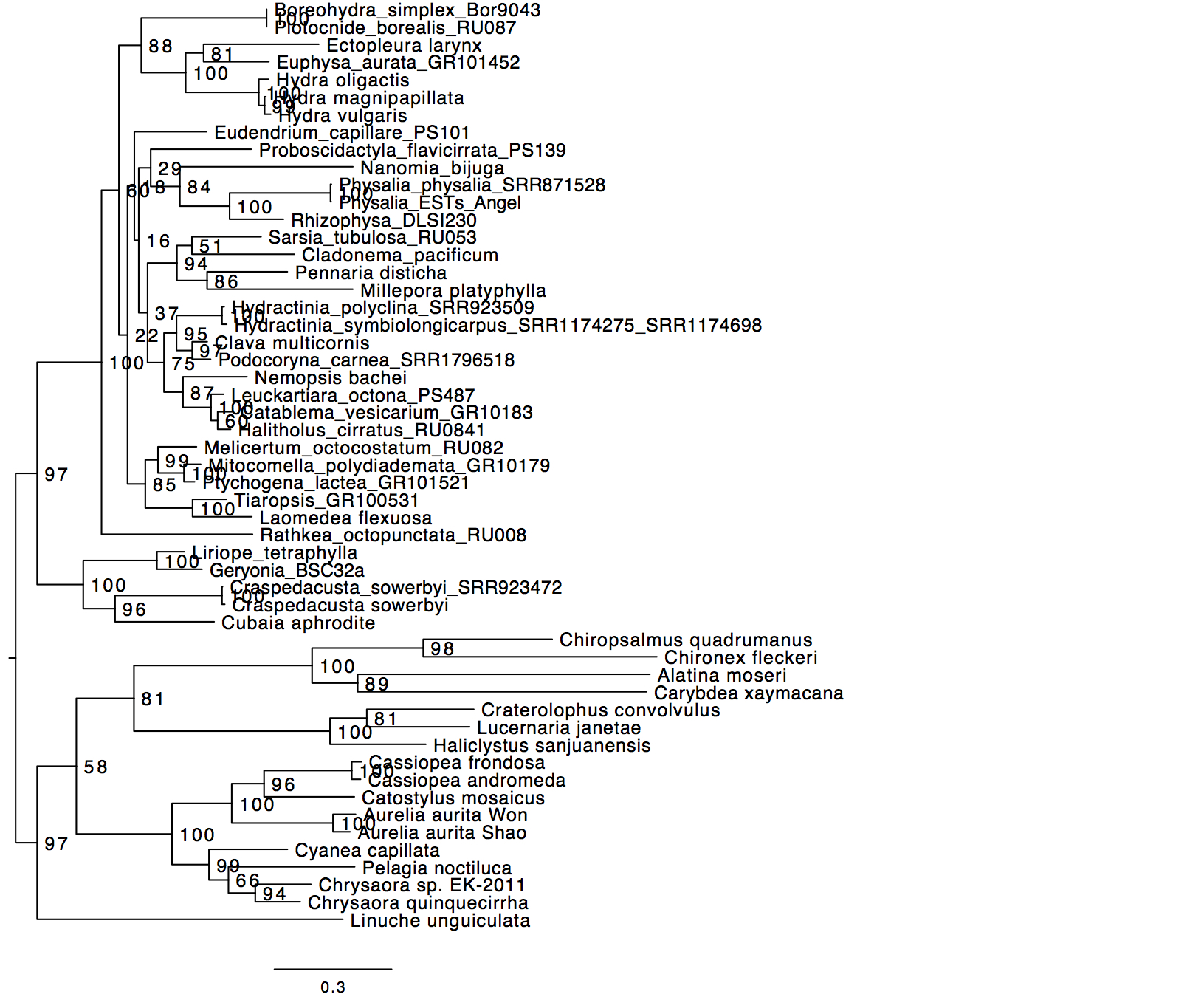

Supplement: Figure S3 — Support values correspond to bootstrap values. [file peerj-03-1403-s003.jpg]

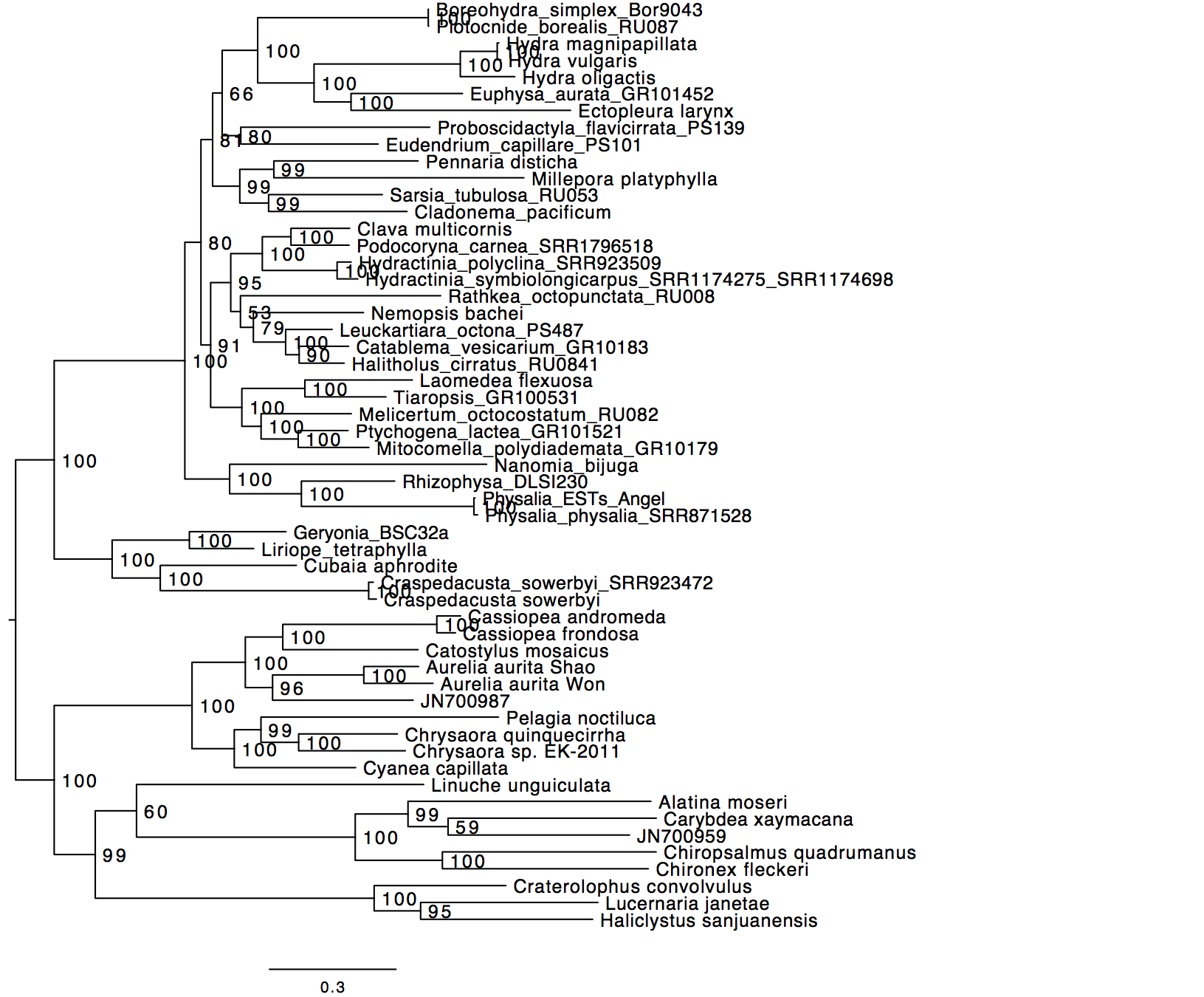

Supplement: Figure S4 — Support values correspond to bootstrap values. [file peerj-03-1403-s004.jpg]

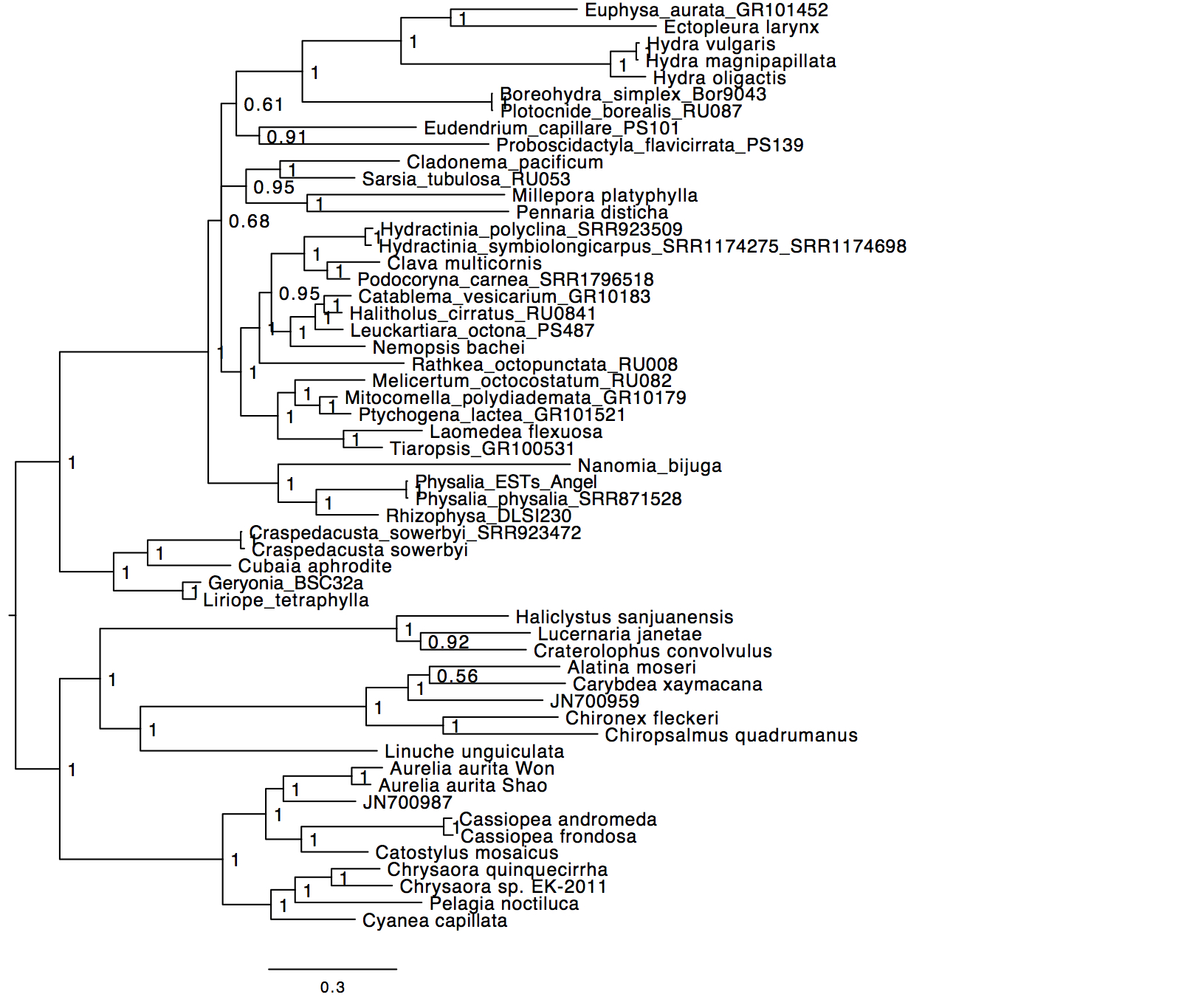

Supplement: Figure S5 — Support values correspond to posterior probabilities. [file peerj-03-1403-s005.jpg]

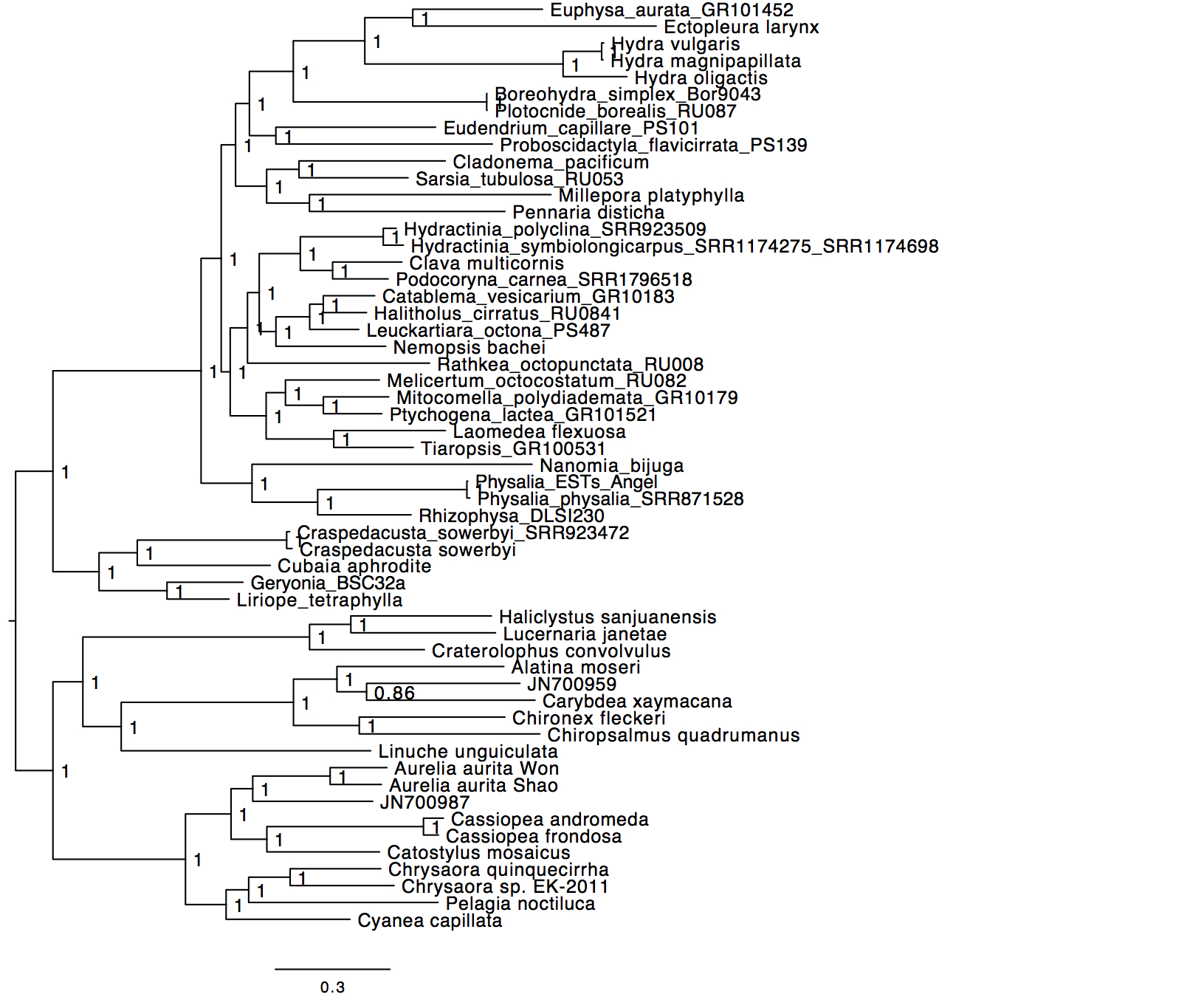

Supplement: Figure S6 — Support values correspond to posterior probabilities. [file peerj-03-1403-s006.jpg]

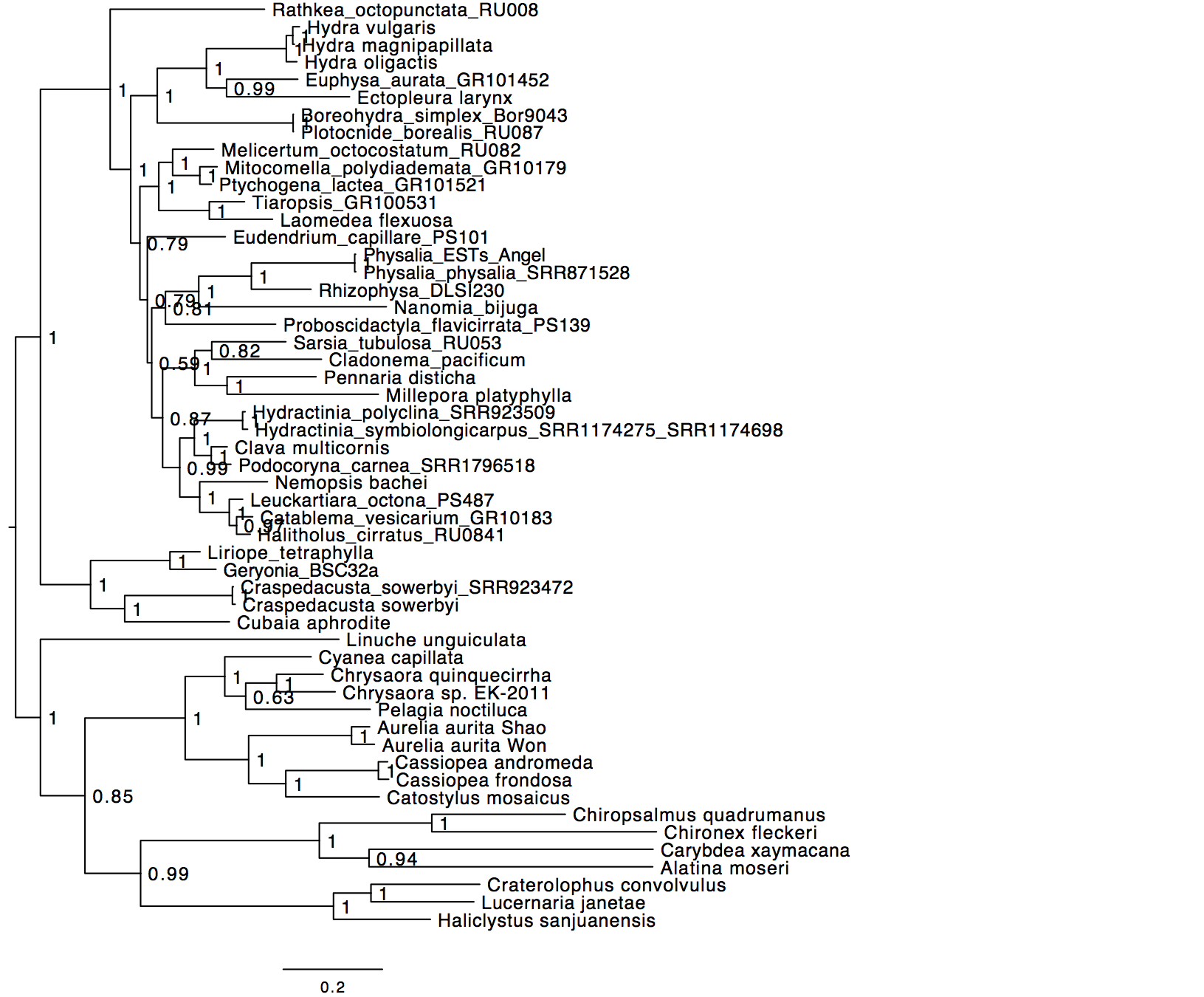

Supplement: Figure S7 — Support values correspond to posterior probabilities. [file peerj-03-1403-s007.jpg]

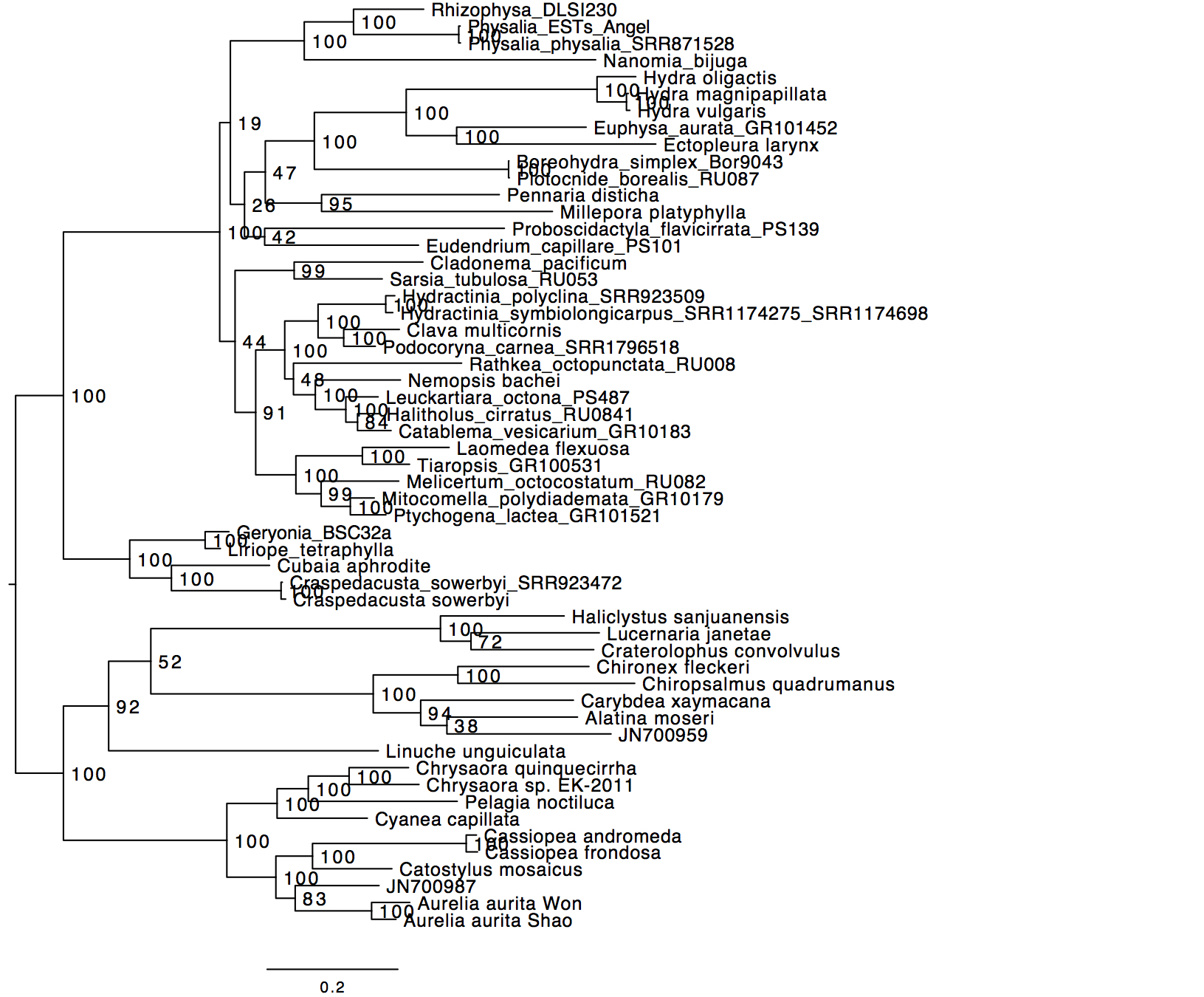

Supplement: Figure S8 — Support values correspond to bootstrap values. [file peerj-03-1403-s008.jpg]

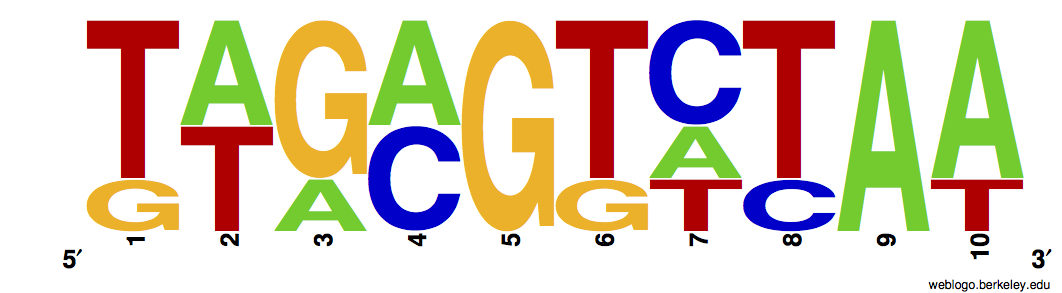

Supplement: Figure S9 — IGRs were aligned using GLAM2 from the MEME suit v. 4.10.1 (meme-suite.org/); the logo was created using WebLogo 3 (weblogo.threeplusone.com/create.cgi). [file peerj-03-1403-s009.jpg]

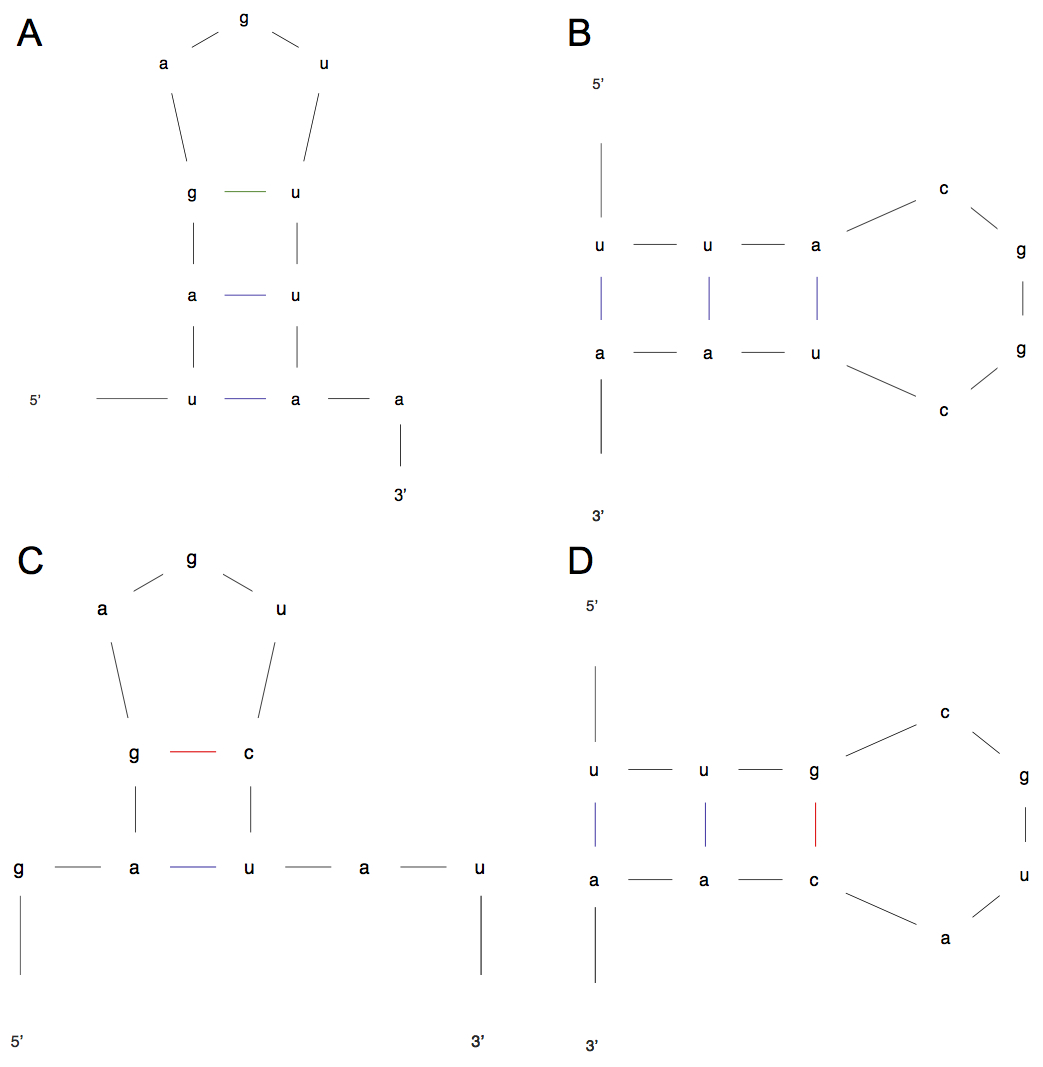

Supplement: Figure S10 [file peerj-03-1403-s010.jpg]
