# Supplementary material for: Phylogenetic analysis of higher-level relationships within Hydroidolina (Cnidaria: Hydrozoa) using mitochondrial genome data and insight into their mitochondrial transcription
Source: PeerJ. 2015 Nov 19;3:e1403. doi: 10.7717/peerj.1403 (PMC4655093; doi:10.7717/peerj.1403)
Supplement: Table S1 — HYDR-rnl-R1 TTTAAAGGTCGAACAGACCTACC [file peerj-03-1403-s011.pdf]

Species	Primer left	Primer right	PCR size
Boreohydra simplex	HYDR-rnl-R1	cox1-r	>12 kb
Catablema vesicarium	HYDR-rnl-R1	cox1-r	>13 kb
Eudendrium capillare	rnl-r	nad5-f	6 kb
	nad5-f	cox1-r	>7 kb
Euphysa aurata	HYDR-rnl-F1	cox1-r	>12 kb
Geryonia proboscidalis	cox1-r	cob-r	>11 kb
Halitholus cirratus	HYDR-rnl-R1	cox1-r	>13 kb
Leuckartiara octona	HYDR-rnl-R1	cox1-r	>13 kb
Melicertum octocostatum	HYDR-rnl-R1	cox1-r	>13 kb
Mitrocomella polydiademata	HYDR-rnl-R1	cox1-r	>13 kb
Plotocnide borealis	HYDR-rnl-F1	cox1-r	>12 kb
Proboscidactyla flavicirrata	nad5-f	cob-r	6 kb
Ptychogena lactea	HYDR-rnl-R1	cox1-r	>13 kb
Rathkea octopunctata	HYDR-rnl-R1	cox1-r	>13 kb
Rhizophysa eysenhardti	cox1-r	cox1-r	>13 kb
Sarsia tubulosa	HYDR-rnl-R1	cox1-r	>13 kb
Tiaropsis multicirrata	HYDR-rnl-R1	cox1-r	>13 kb
