# Supplementary material for: Phylogenetic analysis of higher-level relationships within Hydroidolina (Cnidaria: Hydrozoa) using mitochondrial genome data and insight into their mitochondrial transcription
Source: PeerJ. 2015 Nov 19;3:e1403. doi: 10.7717/peerj.1403 (PMC4655093; doi:10.7717/peerj.1403)
Supplement: Table S2 — Iss.cSym critical Iss value for symetrical tree; Asym critical Iss value for completely assymetrical tree. Bold values correspond to saturation levels rendering the sequences useless for phylogenetics. [file peerj-03-1403-s012.pdf]

Alignments		4 OTUs	8 OTUs	16 OTUs	32 OTUs
NT	Iss	0.588	0.589	0.618	0.649
	Iss.cSym	0.857	0.845	0.848	0.817
	P	0.000	0.000	0.000	0.000
	Iss.cAsym	0.846	0.763	0.677	**0.571**
	P	0.000	0.000	0.000	**0.000**
rRNA	Iss	0.680	0.704	0.750	0.832
	Iss.cSym	0.842	0.825	0.809	0.793
	P	0.000	0.000	0.005	0.112
	Iss.cAsym	0.818	0.732	**0.637**	**0.524**
	P	0.000	0.107	**0.000**	**0.000**
allnt	Iss	0.597	0.598	0.647	0.695
	Iss.cSym	0.858	0.844	0.851	0.818
	P	0.000	0.000	0.000	0.000
	Iss.cAsym	0.847	0.761	0.675	**0.572**
	P	0.000	0.000	0.000	**0.000**
