# Supplementary material for: Phylogenetic analysis of higher-level relationships within Hydroidolina (Cnidaria: Hydrozoa) using mitochondrial genome data and insight into their mitochondrial transcription
Source: PeerJ. 2015 Nov 19;3:e1403. doi: 10.7717/peerj.1403 (PMC4655093; doi:10.7717/peerj.1403)
Supplement: Table S3 [file peerj-03-1403-s013.pdf]

Name	Size	Ambiguities	Informative sites		Percentage	
			with gaps	without gaps	with gaps	without gaps
AA	2902	0	2501	1851	14	36
NT	9864	205	8850	7074	10	28
rRNA	2154	17	1664	1426	23	34
allNT	12018	222	10773	8500	10	29
