# Supplementary material for: Phylogenetic analysis of higher-level relationships within Hydroidolina (Cnidaria: Hydrozoa) using mitochondrial genome data and insight into their mitochondrial transcription
Source: PeerJ. 2015 Nov 19;3:e1403. doi: 10.7717/peerj.1403 (PMC4655093; doi:10.7717/peerj.1403)
Supplement: Table S4 — /3Phos/3’ Phosphorylation; * Phosphorothioated DNA base [file peerj-03-1403-s014.pdf]

Name	Sequence
UniAdp_barco1	ACACTCTTTCCCTACACGACGCTCTTCCGATCTCAGG*T*T/3Phos/
UniAdp_barco2	ACACTCTTTCCCTACACGACGCTCTTCCGATCTGCTA*T*T/3Phos/
UniComp_barco1	AACCTGAGATCGGAAGAGC
UniComp_barco2	AATAGCAGATCGGAAGAGC
IndAdp_short	GTGACTGGAGTTCAGACGTGTGCTCTTCCGAT*C*T/3Phos/
IndCompAdp	AGATCGGAAGAGCA
UniAdp_long	AATGATACGGCGACCACCGAGATCTACACTCTTTCCCTACACGACGC
UniPrim1_PCR	ACACTCTTTCCCTACACGACGC
UniPrim2_PCR	AATGATACGGCGACCACCGAGATC
IndPrim1_PCR	GTGACTGGAGTTCAGACGTG
IndPrim2_PCR	CAAGCAGAAGACGGCATACGAG
IndAdp_long1	CAAGCAGAAGACGGCATACGAGATTCGCAGGGTGACTGGAGTTCAGACGTG
IndAdp_long2	CAAGCAGAAGACGGCATACGAGATCTCTGCAGTGACTGGAGTTCAGACGTG
IndAdp_long3	CAAGCAGAAGACGGCATACGAGATCCTAGGTGTGACTGGAGTTCAGACGTG
IndAdp_long4	CAAGCAGAAGACGGCATACGAGATGGATCAAGTGACTGGAGTTCAGACGTG
IndAdp_long5	CAAGCAGAAGACGGCATACGAGATGCAAGATGTGACTGGAGTTCAGACGTG
IndAdp_long6	CAAGCAGAAGACGGCATACGAGATATGGAGAGTGACTGGAGTTCAGACGTG
IndAdp_long7	CAAGCAGAAGACGGCATACGAGATCTCGATGGTGACTGGAGTTCAGACGTG
IndAdp_long8	CAAGCAGAAGACGGCATACGAGATGCTCGAAGTGACTGGAGTTCAGACGTG
IndAdp_long9	CAAGCAGAAGACGGCATACGAGATACCAACTGTGACTGGAGTTCAGACGTG
IndAdp_long10	CAAGCAGAAGACGGCATACGAGATCCGGTACGTGACTGGAGTTCAGACGTG
IndAdp_long11	CAAGCAGAAGACGGCATACGAGATAACTCCGGTGACTGGAGTTCAGACGTG
IndAdp_long12	CAAGCAGAAGACGGCATACGAGATTTGAAGTGTGACTGGAGTTCAGACGTG
IndAdp_long13	CAAGCAGAAGACGGCATACGAGATACTATCAGTGACTGGAGTTCAGACGTG
IndAdp_long14	CAAGCAGAAGACGGCATACGAGATTTGGATCGTGACTGGAGTTCAGACGTG
IndAdp_long15	CAAGCAGAAGACGGCATACGAGATCGACCTGGTGACTGGAGTTCAGACGTG
IndAdp_long16	CAAGCAGAAGACGGCATACGAGATTAATGCGGTGACTGGAGTTCAGACGTG
IndAdp_long17	CAAGCAGAAGACGGCATACGAGATAGGTACCGTGACTGGAGTTCAGACGTG
IndAdp_long18	CAAGCAGAAGACGGCATACGAGATTGCGTCCGTGACTGGAGTTCAGACGTG
IndAdp_long19	CAAGCAGAAGACGGCATACGAGATGAATCTCGTGACTGGAGTTCAGACGTG
IndAdp_long20	CAAGCAGAAGACGGCATACGAGATCATGCTCGTGACTGGAGTTCAGACGTG
