# Supplementary material for: Phylogenetic analysis of higher-level relationships within Hydroidolina (Cnidaria: Hydrozoa) using mitochondrial genome data and insight into their mitochondrial transcription
Source: PeerJ. 2015 Nov 19;3:e1403. doi: 10.7717/peerj.1403 (PMC4655093; doi:10.7717/peerj.1403)
Supplement: Table S5 [file peerj-03-1403-s015.pdf]

Null Hypothesis	Topological Constraint	AU Test p-value			
		AA	NT	allNT	rRNA
Aplanulata 1st	(Outgroup (Trachylina (Aplanulata (Siphonophora, Capitata, Leptothecata, Filifera I, Filifera II, Filifera III, Filifera IV))))	0.359	3.00E-004	2.00E-004	0.53
Capitata 1st	(Outgroup (Trachylina (Capitata (Aplanulata, Siphonophora, Leptothecata, Filifera I, Filifera II, Filifera III, Filifera IV))))	0.365	0.001	2.00E-004	0.13
Siphonophora 1st	(Outgroup (Trachylina (Siphonophora (Aplanulata, Capitata, Leptothecata, Filifera I, Filifera II, Filifera III, Filifera IV))))	0.687	1	1	0.617
Monophyly of Filifera	(Outgroup (Trachylina (Siphonophora, Aplanulata, Capitata, Leptothecata (Filifera I, Filifera II, Filifera III, Filifera IV))))	0.002	5.00E-012	1.00E-007	0.738
Polyphyly of Filifera	(Outgroup (Trachylina (Siphonophora, Capitata (Aplanulata, Filifera I, Filifera II) (Leptothecata, Filifera III, Filifera IV))))	0.998	1	1	0.262
Monophyly of Anthoathecata	(Outgroup (Trachylina (Siphonophora, Leptothecata (Capitata, Aplanulata, Leptothecata, Filifera I, Filifera II, Filifera III, Filifera IV))))	0.096	0.014	0.003	0.715
Polyphyly of Anthoathecata	(Outgroup (Trachylina (Siphonophora (Capitata, Aplanulata, Filifera I, Filifera II) (Leptothecata, Filifera III, Filifera IV))))	0.904	0.986	0.997	0.285
